# Health literacy scale for English-speaking children: translation and validation of the HLS-Child-Q15-EN

**DOI:** 10.1136/bmjopen-2025-110215

**Published:** 2025-12-17

**Authors:** Thomas Frazer Eagle Drake-Brockman, Vance Locke, Neil Hauser, David Sommerfield, Daisy Evans, Aine Sommerfield, Nazim Khan, Britta Sylvia von Ungern-Sternberg

**Affiliations:** 1 Institute for Paediatric Perioperative Excellence, The University of Western Australia, Perth, Western Australia, Australia; 2 Anaesthesia and Pain Medicine, Perth Children’s Hospital, Child and Adolescent Health Service, Nedlands, Western Australia, Australia; 3 Medical School, The University of Western Australia, Perth, Western Australia, Australia; 4 Perioperative Medicine Team, The Kids Research Institute Australia, Nedlands, Western Australia, Australia; 5 School of Psychology, The University of Western Australia, Perth, Western Australia, Australia; 6 School of Physics, Maths and Computing, The University of Western Australia, Perth, Western Australia, Australia

**Keywords:** PAEDIATRICS, Child, Health Education, Health Literacy

## Abstract

**Abstract:**

**Objective:**

To translate and validate the HLS-Child-Q15, a relatively short questionnaire for assessing health literacy in children originally validated in German, into English to make it accessible to a large population of English-speaking children.

**Design:**

We translated the HLS-Child-Q15 into English following established methods, including forward and backward translation, using multiple translators. We incorporated clinician and consumer input into the translation process. We conducted a qualitative pre-test to assess comprehension and a validation to assess psychometric properties and test-retest reliability.

**Setting:**

Perth Children’s Hospital, Perth, Western Australia

**Patients:**

We recruited English-speaking children aged 8 to 15 years.

**Main outcome measures:**

Qualitative analysis of pre-test interviews, Cronbach’s α coefficient for internal consistency and intraclass correlation coefficient for test-retest reliability.

**Results:**

The translation process yielded an acceptable translation. A qualitative pre-test conducted with 10 children demonstrated good comprehension of questionnaire items and resulted in small changes to increase item clarity. Validation with 207 participants demonstrated that questionnaire output score increased with age, school year, self-efficacy score, parental educational level and home literacy environment score. Internal consistency was assessed, with a Cronbach’s α coefficient of 0.854 (95% CI 0.812 to 0.887). Test-retest reliability was moderate, with an intraclass correlation coefficient of 0.612 (95% CI 0.402 to 0.761).

**Conclusions:**

The translated HLS-Child-Q15 was well understood by children. Validation of the translated questionnaire demonstrated adequate psychometric properties, consistent with the original German questionnaire. The translated HLS-Child-Q15 is suitable for use with English-speaking children.

**Data availability statement:**

Data are available on reasonable request and in compliance with institutional ethics and governance requirements.

**Trial registration number:**

ACTRN12622001499774

**STRENGTHS AND LIMITATIONS OF THIS STUDY**

- Translation of the original German-language questionnaire was performed with a robust forward and backward translation process using multiple translators.
- Consumer input was integrated at multiple stages to ensure acceptability and a qualitative pre-test was completed to assess comprehension of the items.
- Validation in a cohort of 207 children was completed, along with a test-retest subgroup.
- All children were recruited from a single tertiary centre in Western Australia.

## Introduction

 Health literacy is ‘the skills, knowledge, motivation and capacity of a person to access, understand, appraise and apply information to make effective decisions about health and healthcare and take appropriate action.’^1^ Low health literacy is associated with a wide range of adverse outcomes, including increased hospitalisation and emergency presentations, lower uptake of vaccination, poor health and higher risk of death among older people.^2^ Across Europe, rates of high health literacy in children are low, from 13% to 38%, with most having moderate health literacy.^3^ Assessing health literacy is useful in clinical practice and health research, guiding interventions in health literacy itself, and tailoring education.

The HLS-Child-Q15 is a health literacy tool specifically developed for children consisting of 15 short questions. It is relatively short, simple and quick to complete and does not require significant numerical abilities.^4 5^ Each item asks how difficult or easy the child finds a particular health task (eg,‘find out which food is healthy for you?’) with a score from 1 for ‘very difficult’ to 4 for ‘very easy’.

The tool is validated in German (ages 8–12, but 92.8% age 9–10), Dutch (ages 8–11) and French (ages 8–11), with all versions demonstrating high internal consistency.^4 6 7^ A known limitation is that attempts to find an underlying factor structure that aligns with theoretical frameworks of health literacy have been unsuccessful.^4^

While an English translation of the items was published alongside the initial German version and has been used in one published study,^8^ this translation was not done according to a rigorous process for conceptual equivalence and is not validated. A validated English translation can support use in English-speaking populations.

Therefore, we undertook the translation and validation of the HLS-Child-Q15 into English to provide an English-language child-specific health literacy assessment tool for clinical and research applications.

## Methods

This translation and validation study was conducted at Perth Children’s Hospital (PCH), Perth, Australia.

### Patient and public involvement

Consumers of all ages were involved from the conceptualisation and design of the study throughout all phases of the project. The need for this study was a consequence of the design of two other studies that sought to investigate key consumer priorities in paediatric anaesthesia.^9 10^ Consumer priorities, therefore, indirectly drove the objectives of this study. Members of the Anaesthesia Consumer Research Reference Panel, a diverse group of adults who either have personal lived experience in perioperative care during their childhood or are parents/carers of children with lived perioperative experience, provided advice on the study concept and design. Panel members were from diverse socioeconomic backgrounds and also included First Nation Members and persons from culturally and linguistically diverse backgrounds (CaLD). Youth Consumer Ambassadors (aged 6–17 years) were also involved in the conceptualisation and conduct of the study, including review of patient-facing documents. Additionally, a Consumer Buddy was allocated for more intensive review of the study, and five young consumer representatives participated directly in reviewing final item wordings following the qualitative pre-test. Our Research Reference Panel and Consumer Buddy receive participation payments in line with institutional requirements. Study participants who requested a copy of the results of the study will receive a suitable summary targeted to a consumer audience following publication.

### Translation

Our translation methods are based on those published by Sousa & Rojjanasrirat.^11^ The original German-language items of the HLS-Child-Q15 were translated to English by a health professional who is not only fluent in German but also a native English speaker, as per guidelines for the translation of instruments.^11^ The translator was not provided with the previously published English translation and was instructed to aim for conceptual equivalence rather than literal word-for-word translation.

We assembled a panel of research team members, including native English and native German speakers and three bilingual children to review the translations and identify and resolve any inadequate expressions or inconsistencies. We also compared our English translation to the translated items reported in the English-language publication of the original German HLS-Child-Q15. Discrepancies were resolved by consensus.

This initial consensus translation was then back-translated from English to German by two health professionals who are native German speakers to ensure conceptual integrity was retained. Discrepancies from the original items were re-examined to determine if the translated items required revision.

### Qualitative pre-test

To ensure that the translated questionnaire was suitable for the target population, we conducted a qualitative pre-test and cognitive debrief from April to June 2023.

We recruited children aged 8–15 years, stratified equally into two age cohorts, 8 to<12 years and 12–15 years, from the Surgical Short Stay Unit and hospital wards at PCH. A sample size of 10 was chosen for pragmatic reasons, given the available resources. We excluded children if they or their parents were unable to understand sufficient English to agree to participate in the study. No incentives were offered for participation.

Following written informed consent from parents and verbal or written agreement from children, as deemed appropriate by the researcher, participants completed a semi-structured interview, lasting approximately 15 min, conducted by a trained member of the research team (TFED-B) and audio-recorded. The interview included demographics for children (age, self-reported gender), administration of the translated HLS-Child-Q15 health literacy items, and debriefing for each item. The time taken for each child to complete the translated HLS-Child-Q15 was recorded. There was no other activity or follow-up.

Descriptive statistics were obtained for demographics and the time taken to administer the tool. Interview recordings were transcribed verbatim and subject to thematic analysis by three analysts (TFED-B, a student and a research assistant) using the Framework Method and NVivo software.^12^ Initially, analysts familiarised themselves with the transcripts and then established a set of agreed deductive (each item of the questionnaire, the question stem and response options) and inductive codes identified during the familiarisation. All three analysts then coded the same three transcripts, and codings were compared and discussed. The remaining transcripts were then coded by a single analyst. During this process, the team met to clarify meanings and ensure consistency. The coded transcripts were then charted onto a Framework Matrix by the same analyst team, and each code was summarised. The results were used to assess the items of the translated questionnaire and to make any necessary modifications to the items.

### Validation

Following translation and qualitative pre-testing, the tool underwent validation of its psychometric properties and test-retest reliability, with recruitment taking place from May 2024 to March 2025.

The sample size was determined with reference to guidelines for scale validation, having at least 10 participants per survey item, or a minimum sample size of 200.^13^ We planned to recruit 200 children aged 8–15 years, stratified equally into two age cohorts, 8 to<12 years and 12–15 years, from all areas of our tertiary institution. We excluded children if they or their parents could not understand sufficient English to agree to inclusion in the study. No incentives were offered for participation.

Participants who completed the initial survey could opt in to a retest component. While the intraclass correlation coefficient (ICC) of the HLS-Child-Q15 test-retest is unknown, we conservatively estimated that if 30 survey respondents completed the follow-up survey, a 90% power to detect an ICC of 0.5 would be achieved.

Recruitment was by online questionnaire with REDCap eConsent,^14^ and families in the hospital could self-enrol using a QR code displayed in waiting areas. We also directly approached families to promote the study. The online survey was configured to close after 200 participants successfully completed the full questionnaire, and therefore, participants who did not complete the HLS-Child-Q15-EN questions were replaced.

The questionnaire collected demographics (age and self-reported gender), school year, primary caregiver’s highest education attainment, the Home Literacy Environment (HLE) single-item measure (number of children’s books in the home) and each item of the HLS-Child-Q15-EN. Three questions designed to measure self-efficacy (‘I can trust in my knowledge and abilities,’ ‘I can find a solution for most problems’ and ‘If I make an effort, I will succeed’, measured on a four-point scale of agreement) were also included.

Participants who opted into the retest follow-up were automatically sent a link after 2 weeks to complete the follow-up questionnaire, which contained the HLS-Child-Q15-EN only. There was no other activity or further follow-up.

Descriptive statistics were obtained for demographics and HLS-Child-Q15-EN items. For each item, the proportion of responses in each category was computed. To assess comprehension of the HLS-Child-Q15-EN items, the proportion of ‘I don’t know’ responses was calculated using data from the qualitative pre-test, as this option was not presented in the validation questionnaire.^6^

Exploratory factor analysis was performed on the HLS-Child-Q15-EN to determine if an underlying factor structure could be identified. Cronbach’s α was calculated to assess the internal consistency of the scale.^13^ We predetermined that a value of 0.70 or greater was acceptable. Test-retest reliability was assessed by calculating the ICC, where an ICC closer to 1.0 indicated greater scale reliability.^15^An exploratory principal components analysis (PCA) was also conducted, including plots of the principal components (PCs) to facilitate interpretation.

The HLS-Child-Q15-EN output score was calculated in the same manner as the original HLS-Child-Q15 (referred to as ‘HL mean score’) as the mean of item scores when at least 12 of 15 items were answered. A linear regression model was fitted to the data with the HLS-Child-Q15-EN output score as the outcome and participant demographics (age, gender, primary caregiver’s highest educational attainment and HLE) as predictors to assess differentiation between known groups, an aspect of scale validity.^13^ We expected that the HLS-Child-Q15-EN output scores would be higher for children who are older, who had higher HLE scores or whose parents had higher educational attainment. We expected the score to be independent of gender. To quantify discriminant validity, we calculated Spearman’s rank-order correlation coefficient between the HLS-Child-Q15-EN output score and the self-efficacy score; a weak to moderate correlation coefficient would indicate that the HLS-Child-Q15-EN and self-efficacy scores measure distinct concepts.

Ethics approval was received from the Child and Adolescent Health Service Human Research Ethics Committee (RGS0000005719), recognised by the University of Western Australia Human Research Ethics Committee (2022/ET000899), and was registered prospectively with ANZCTR (ACTRN12622001499774).

## Results

### Translation

The original items of the German HLS-Child-Q15 were independently translated by two doctors, who were native English speakers and fluent in German. Minor discrepancies were resolved by consensus of the research team. Three bilingual children also assessed the translations. One item was modified to reference ‘crossing the road’ instead of ‘traffic safety lessons’ as this was felt to be culturally specific. We also substituted ‘keep your weight healthy’ instead of ‘don’t get too fat or too thin’ as the original wording would have been controversial. Back translation by two other doctors who were native German speakers and fluent in English demonstrated acceptable alignment to the original German items.

### Qualitative pre-test

We approached 13 children and recruited and interviewed 10 (78%). Demographics are presented in table 1. The mean (range) time taken to administer the provisionally translated HLS-Child-Q15 was 4.7 (3.2–8.0) min. The researcher conducting the interviews was satisfied that saturation, the point at which no new insights were emerging, was reached. The children interviewed could answer the provisionally translated items and reported that they understood the questions. Most children were able to paraphrase the questions to an acceptable level, and a few suggestions for change were made.

**Table 1:** Demographics of children who participated in qualitative pre-test interviews and validation of the proposed HLS-Child-Q15-EN questionnaire

Characteristic	Overall	Stratum 1	Stratum 2
Qualitative pre-test			
n	10	5	5
Gender			
Man or male (boy)	8 (80%)	4 (80%)	4 (80%)
Woman or female (girl)	2 (20%)	1 (20%)	1 (20%)
Non-binary	0 (0%)	0 (0%)	0 (0%)
They use a different term	0 (0%)	0 (0%)	0 (0%)
Prefer not to answer	0 (0%)	0 (0%)	0 (0%)
Age	11.5 (1.509)	10.2 (0.447)	12.8 (0.837)
Validation			
n	207	105	102
Gender			
Man or male (boy)	98 (47%)	57 (54%)	41 (40%)
Woman or female (girl)	108 (52%)	48 (46%)	60 (59%)
Non-binary	0 (0%)	0 (0%)	0 (0%)
They use a different term	0 (0%)	0 (0%)	0 (0%)
Prefer not to answer	1 (0.5%)	0 (0%)	1 (1.0%)
Age	11.5 (2.3)	9.5 (1.2)	13.5 (1.1)
School Year			
Does not attend school	0 (0%)	0 (0%)	0 (0%)
Year 1–2	5 (2.4%)	5 (4.8%)	0 (0%)
Year 3–4	45 (22%)	45 (43%)	0 (0%)
Year 5–6	58 (28%)	50 (48%)	8 (7.8%)
Year 7–8	52 (25%)	5 (4.8%)	47 (46%)
Year 9–10	45 (22%)	0 (0%)	45 (44%)
Year 11–12	2 (1.0%)	0 (0%)	2 (2.0%)
Caregiver’s Highest Educational Attainment			
Lower (None, Primary, Year 9)	17 (8.2%)	7 (6.7%)	10 (9.8%)
Secondary/Certificate (Year 10–12, Cert I-IV)	58 (28%)	29 (28%)	29 (28%)
Tertiary (Dip/AdvDip, Bachelor’s Degree)	73 (35%)	37 (35%)	36 (35%)
Higher (Grad Cert/Dip, Postgrad Degree)	59 (29%)	32 (30%)	27 (26%)
HLE	75 (30, 150)	100 (50, 150)	55 (25, 100)

Values are count (proportion), mean (SD), or median (Q1, Q3)

HLE, Home Literacy Environment (number of children’s books in the home).

Item 11 was answered well, but most children had some difficulty with the phrases ‘figure out’ and ‘help you a lot or a little to stay healthy.’ In particular, it was unclear if the latter phrase is seeking the child to consider one or two categories of things. Consensus was reached on replacing these phrases with ‘decide which’ and ‘more healthy or less healthy’ to increase clarity.

Item 15 was initially translated using the phrase ‘to eat a healthy diet’; however, some children considered ‘diet’ to mean a restrictive eating rather than nutritional balance. We replaced ‘healthy diet’ with ‘healthy food’ to make this clearer and align with phrasing used by school educators.

The response options were initially translated as ‘very difficult’, ‘fairly difficult’, ‘fairly easy’ and ‘very easy.’ While only commented on by one child, it was apparent in interviews that ‘fairly’ was challenging for some children. After considering ‘little’ and ‘a bit’, we settled on ‘a bit.’ Many children wanted a central or neutral response; however, we felt this would be an unwanted deviation from the original HLS-Child-Q15, and that forcing a non-neutral response was an intended property of the original scale. Participants may use a neutral option in a variety of ways, including as an opt-out or a ‘dumping ground’ when unfamiliar with the content, which may be undesirable.^16 17^

We sought direct feedback from five young consumer representatives, who suggested a number of tweaks to word order and grammar to improve comprehension. The team considered these suggestions to achieve consensus on the final item wording (online supplemental table S1).

### Validation

**Figure 1:**
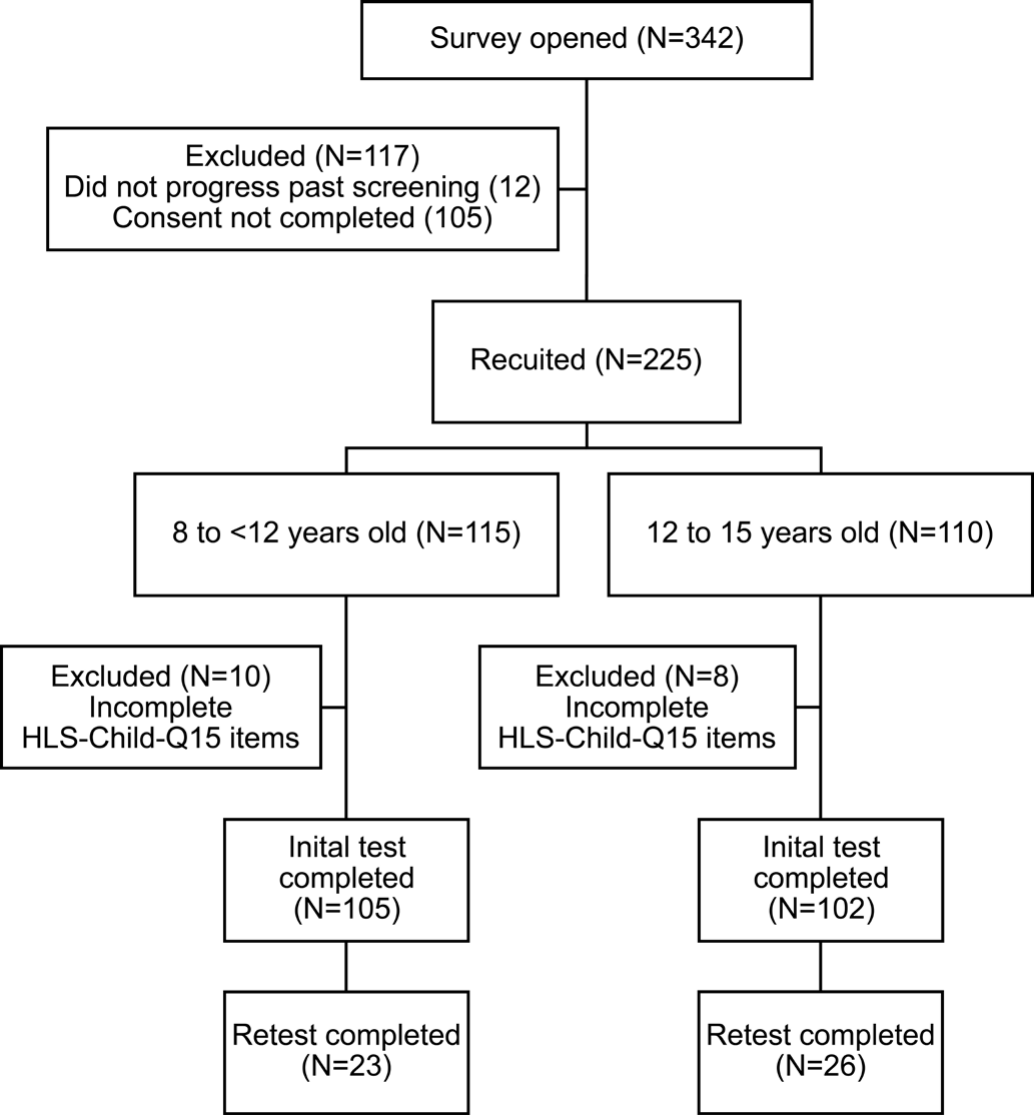
Participant flow diagram for the validation of the proposed HLS-Child-Q15-EN questionnaire.

Total recruitment was 207, exceeding our planned recruitment due to an error in the stopping condition checks in our REDCap survey. After careful consideration, we chose to include all the records in the analysis. The retest questionnaire was completed by 49 (24%) participants, exceeding our conservative estimate of 30. A participant flow diagram is presented in figure 1 and demographics in table 1.

Item analysis is presented in table 2. It was not possible to skip questions in our instrument, so there were no missing values. The ‘Don’t Know’ was offered in the researcher-administered qualitative pre-test and was only recorded for Item 2 for a single child. This option was not offered in the self-administered validation survey. Proportion of maximum agreement (‘Very Easy’ response) ranged between 23% (Item 6) and 81% (Item 14). The SD of responses ranged between 0.568 and 0.955.

**Table 2:** Item analysis for validation of the translated HLS-Child-Q15 questionnaire. Factor analysis produces a three-factor model

Item	How easy or difficult is it for you to…	Score			Maximum agreement	Factor		
		Overall	Stratum 1	Stratum 2		1	2	3
1	Find out how to get better from a cold quickly?	3.014 (0.791)	2.857 (0.802)	3.176 (0.750)	60 (29%)	0.416	–	0.278
2	Find out what you can do to keep a healthy weight?	3.227 (0.808)	3.210 (0.817)	3.245 (0.801)	90 (43%)	0.536	0.198	0.332
3	Find out how to relax best?	3.256 (0.817)	3.257 (0.866)	3.255 (0.767)	95 (46%)	0.101	0.493	0.155
4	Find out which food is healthy for you?	3.348 (0.747)	3.295 (0.746)	3.402 (0.748)	103 (50%)	0.267	0.208	0.938
5	Understand when and how to take your medications when you are sick?	3.213 (0.844)	3.114 (0.880)	3.314 (0.796)	95 (46%)	0.470	–	0.106
6	Understand what a doctor is saying to you?	2.889 (0.796)	2.886 (0.824)	2.892 (0.770)	48 (23%)	0.521	0.268	0.140
U7	understand why you need to see a doctor sometimes even though you are not sick?	3.082 (0.852)	3.038 (0.876)	3.127 (0.829)	77 (37%)	0.383	0.245	–
8	Understand why you should have vaccinations?	3.280 (0.955)	3.143 (0.965)	3.422 (0.927)	114 (55%)	0.247	0.444	–
9	Understand what your parents explain to you about your health?	3.396 (0.736)	3.429 (0.691)	3.363 (0.781)	109 (53%)	0.661	0.305	–
10	Understand why you need to rest sometimes?	3.415 (0.789)	3.286 (0.829)	3.549 (0.726)	120 (58%)	–	0.772	0.125
11	Decide which things are more healthy or less healthy?	3.353 (0.722)	3.343 (0.718)	3.363 (0.728)	102 (49%)	0.396	0.314	0.399
12	Do what your parents tell you when you are sick so you can get better?	3.343 (0.838)	3.314 (0.870)	3.373 (0.807)	112 (54%)	0.412	0.523	0.117
13	Take your medicine as you are told?	3.425 (0.802)	3.305 (0.911)	3.549 (0.654)	123 (59%)	0.346	0.372	–
14	Remember the rules when you are crossing the road?	3.754 (0.568)	3.638 (0.681)	3.873 (0.390)	168 (81%)	0.237	0.350	0.254
15	Eat healthy food?	3.251 (0.791)	3.219 (0.784)	3.284 (0.801)	90 (43%)	0.538	0.195	0.258
HLS-Child-Q15-EN Score		3.283 (0.455)	3.222 (0.446)	3.346 (0.458)				

Item scores are mean (SD), rates of maximum agreement are count (proportion) and factor loadings are given.

**Figure 2:**
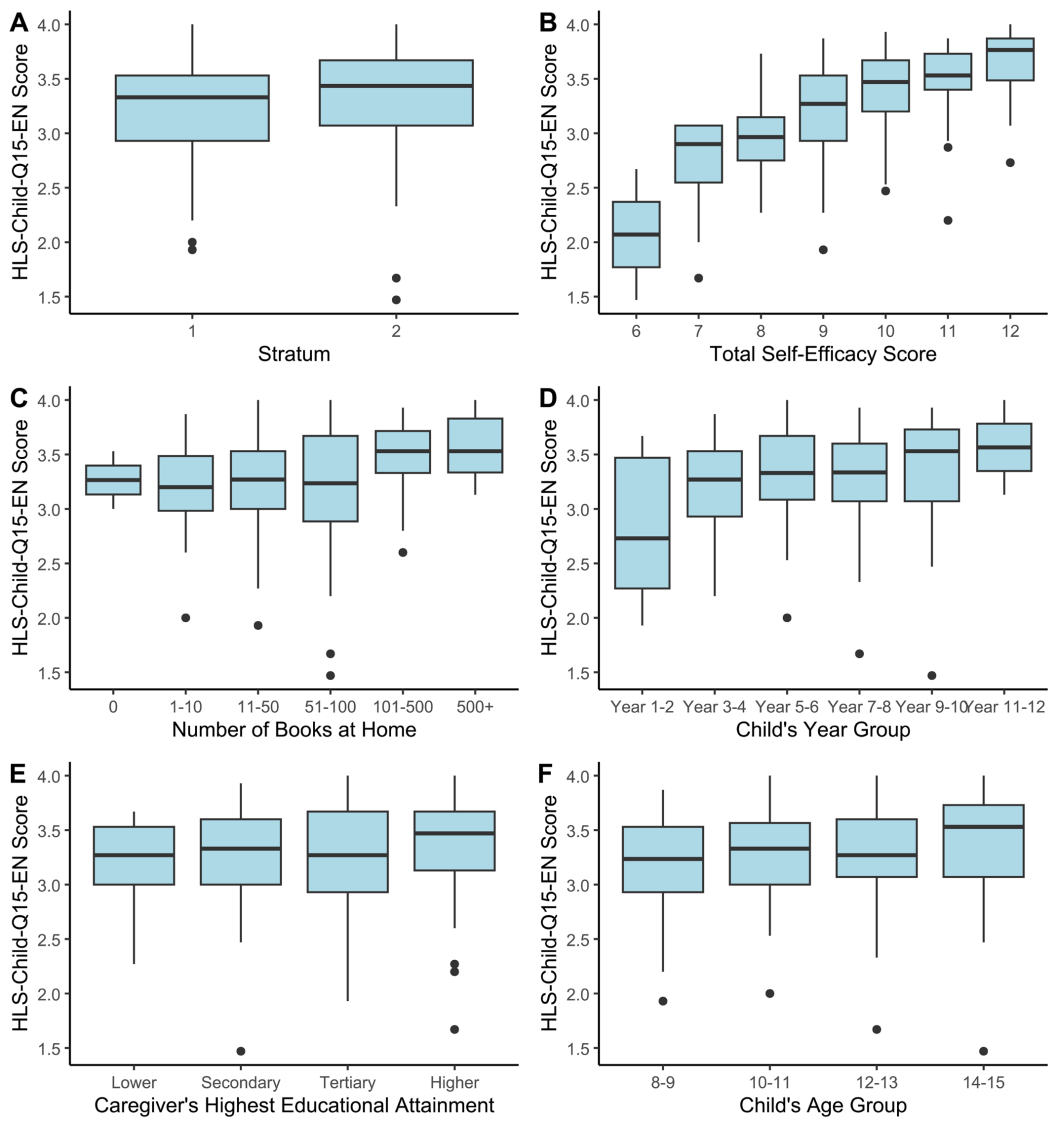
Boxplot of HLS-Child-Q15 scores grouped by self-efficacy question scores and demographic characteristics. Variation in HLS-Child-Q15-EN output score between groups is consistent with expectations in that score increases alongside child self-efficacy (**B**), number of books at home (**C**), age (**F**) and year (**D**) group, and primary carer’s education (**E**).

HLS-Child-Q15-EN output score was greater for Stratum 2 (3.346 vs 3.222) and increased with age, school year, self-efficacy score, parental educational level and HLE (figure 2). There were no significant differences in output scores between boys and girls (3.322 vs 3.256, p=0.295).

Convergent validity was assessed by measuring correlation between HLE and HLS-Child-Q15-EN output scores, which demonstrated a statistically significant but weak positive relationship (r=0.246, p<0.001).

Discriminant validity was examined by comparison with self-efficacy scores (figure 2B). Weak to moderate associations existed between health literacy and self-efficacy, with Spearman’s rank correlations for individual self-efficacy items ranging from ρ=0.333 to 0.462 (p<0.001) and ρ=0.529 (p<0.001) for total self-efficacy score.

Internal consistency was assessed, with an α coefficient (95% CI) of 0.854 (0.812–0.887) indicating satisfactory internal consistency. Test-retest demonstrated moderate reliability, with ICC (95% CI) of 0.612 (0.402–0.761), as seen in online supplemental figure S1. Visual inspection of a Bland-Altman plot demonstrated no significant systematic bias.

Exploratory factor analysis produced three factors with eigenvalues greater than 1, cumulatively accounting for 39.8% of total variance (table 2). The three factors had eigenvalues of 5.093, 1.355 and 1.039, which explained 16.5%, 13.5% and 9.8% of the variance, respectively.

The extracted three-factor model did not suggest a clear conceptual mapping – with the first factor representing a weighted average, and perhaps some suggestion that the second factor represented the agreeability or compliance of the child. Further, the three-factor model produced a test statistic of χ² (63) = 109.64 (p=0.000249), which suggested that it was not sufficient to model the data.

We tested the three-factor ‘Model 2’ (factor 1: items 1–4, factor 2: items 5–11, factor 3: items 12–15) used in the original validation, intended to align with theoretical domains (access, understand and apply). Model fit indices are presented in online supplemental table S2), with slightly lower fit indices for the English version as compared with the German original.

Linear regression was fitted with HLS-Child-Q15-EN output score as the response including age (as continuous), gender, parental education and HLE as covariates. The model was reduced by eliminating non-significant variables. The model contained age and home literacy environment as significant variables (table 3). In the model, the output score increased with age and HLE.

**Table 3:** Linear regression modelling with the proposed HLS-Child-Q15-EN output score as outcome.

Variables	Coefficients	95% CI	P value
(Intercept)	2.830	2.521 to 3.139	<0.001
Age	0.034	0.008 to 0.060	0.013
Home Literacy Environment	0·0005	0.0002 to 0.0009	0.002

PCA showed that the first component captured 34% of the variation in the data, while the next two components captured 9% and 7%, respectively. In the first component, the loadings were all between 0.202 (Item 3) and 0.308 (Item 9), representing a weighted average of the items. The second component is a contrast between items 1, 2, 4, 5, 11, 15 and items 3, 8, 10, 12, 13, 14, with zero-loadings on items 6, 7 and 9. Similar to the second factor in our extracted model, this appears to represent agreeability or compliance.

A plot of the first and second PCs (PC1 and PC2, respectively) is shown in online supplemental figure S2, which demonstrated that PC1 is aligned with self-efficacy. No obvious pattern in relation to gender is observed.

## Discussion

We have successfully translated and validated the HLS-Child-Q15 health literacy questionnaire for use with English-speaking children. Given the relative brevity of the questionnaire and its availability in German, Dutch and French (and similar 22-item and 24-item variants in Nepalese), this is an increasingly popular, practical and accessible tool that is now validated in a much larger cohort of children. Since the tool has only 15 questions and can be administered in about 5 minutes, it may be particularly helpful for clinical screening, when large cohorts need to be assessed, or in self-administered research questionnaires. In fact, the instrument’s attractiveness has already led to its application in populations of English-speaking children; however, this was done with the unvalidated translation included in the original German publication, primarily for the benefit of non-German academic readers, rather than as a validated instrument.

We applied a robust forward and backward translation process, translating the questionnaire for both language and culture, and retaining the intent of the items. We did modify Item 14 substantially – with reference to road safety lessons being replaced with ‘the rules when you are crossing the road’. This was agreed by medical professionals with German background to be a close cultural equivalence. Some other items required notable changes to avoid inadvertent misinterpretation – e.g. in Item 15, we replaced what was originally translated as ‘diet’ with ‘food’ to avoid erroneous suggestion of restrictive dieting. While we believe this best guides English-speaking children towards the intended meaning of the item, we cannot avoid the full impact of different cultural attitudes towards food or language differences.

Like the original, the HLS-Child-Q15-EN attends to health topics that are relevant and familiar to children: healthcare communication, medication, nutrition and healthy behaviours. Our qualitative pre-test confirmed that these questions are well understood.

Only one item (Item 14) had a proportion of maximum agreement (difficulty) that fell outside the 20% to 80% range, which suggests the items are neither too easy nor too hard. In the validation of the original German questionnaire, Item 14 had a difficulty of 82.5% and our translated item has a difficulty of 81%, which suggests that our translation has not changed the difficulty of this item significantly.

Internal consistency was satisfactory, although we note that Cronbach’s α tends to increase with more items, even without improved reliability. Assessment of convergent validity demonstrated a weak relationship with HLE, which is theoretically consistent. Weak to moderate correlation coefficients suggest that the HLS-Child-Q15-EN and self-efficacy scores measure distinct but related concepts. We also noted a conceptually consistent increasing trend with increased age, year group and parental educational level.

Our assessment of test-retest reliability is novel, and we found moderate reliability over a 2-week period. We are cautious about these results, as the majority of children likely completed the initial survey in a healthcare setting and the retest survey at home or otherwise outside of the healthcare environment. Further evaluation of the test-retest reliability of the HLS-Child-Q15-EN could use shorter test-retest periods or standardise the testing environment (e.g. two consecutive clinic appointments).

Factor analysis did not identify any obviously redundant items. We had similar success—or rather lack thereof—in the application of the theoretical three-factor model from the original validation. In our extracted three-factor model, the second factor appeared to represent agreeability or compliance. Principal component analysis found a similar property in PC2. The lack of a factor structure that aligns with a health literacy theoretical framework is consistent with the original scale, and therefore unsurprising. We recommend, therefore, that the HLS-Child-Q15-EN is used as a single output scale. Further exploration of the dimensionality of the scale may be warranted.

Fit indices were slightly lower than but similar to those found for the original scale, which we find reassuring. Linear regression modelling demonstrated that age and HLE were statistically significant predictors of HLS-Child-Q15-EN output score, which are theoretically consistent.

## Limitations

The process of validation is targeted at establishing the internal plausibility of the scale rather than demonstrating correlation with an external standard. We largely adopted the procedure taken in the development of the original HLS-Child-Q15 for the validation of our translation. We placed significant weight on the psychological pre-test regarding the content of the items. The numerical validation focused on the degree to which the items encode meaningful information (e.g. they have variation), are not hopelessly redundant and vary in relation to other variables in a conceptually plausible manner.

A relatively small number of children were recruited for the qualitative pre-test. Further, the pre-test and validation reported were undertaken at a single tertiary paediatric centre in Western Australia. Variations in both English dialect and sociocultural factors may limit application outside of Australian populations. A larger and broader group of children may reveal additional insights. Re-validation in other English-speaking populations should be undertaken.

We did not compare the HLS-Child-Q15-EN with any other health literacy assessment tools. Doing so would be advantageous but is made difficult by the relative lack of such tools and the greater number of items found in other tools. For example, comparison with the REALM-Teen could be considered, for those aged 10 to 19, but this tool contains 66 items. Comparison with measures of other literacies may also be helpful and should also be considered in future studies.

Finally, the ideal validation of this translation would involve comparing the German, Dutch and French versions of the HLS-Child-Q15 with our English translation in populations of multi-lingual children of multiple native languages, although there are obviously challenges in doing so.

## Conclusion

The rigorously translated HLS-Child-Q15-EN demonstrates acceptable qualitative and quantitative properties for use as a health literacy tool in 8- to 15-year-old children. This is a significant advance in the availability of a simple and relatively short tool for assessing health literacy in English-speaking children. The tool can be self-administered with parental assistance, or administered by a third party, and is likely applicable across a wide range of clinical and research settings.

## Supplementary material

10.1136/bmjopen-2025-110215 online supplemental file 1

## Data Availability

Data are available upon reasonable request.
