# Supplementary material for: Health literacy scale for English-speaking children: translation and validation of the HLS-Child-Q15-EN
Source: BMJ Open. 2025 Dec 17;15(12):e110215. doi: 10.1136/bmjopen-2025-110215 (PMC12716569; doi:10.1136/bmjopen-2025-110215)
Supplement: online supplemental file 1 [file bmjopen-15-12-s001.docx]

# Table of Contents

[Supplementary figures 2](#_Toc200053267)

[Figure S1: Scatter plot showing test-retest scores of children completing the proposed HLS-Child-Q15-EN questionnaire with a 2 week interval 2](#_Toc200053268)

[Figure S2: PCA biplot of the first and second principal components 3](#_Toc200053269)

[Supplementary tables 4](#_Toc200053270)

[Table S1: Items of the HLS-Child-Q15-EN after translation, qualitative pre-test, and consensus process by the research team 4](#_Toc200053271)

[Table S2: Comparison of model fit indices for a three-factor model aligned to theoretical domains for the HLS-Child-Q15 (German) and HLS-Child-Q15-EN 5](#_Toc200053272)

# Supplementary figures


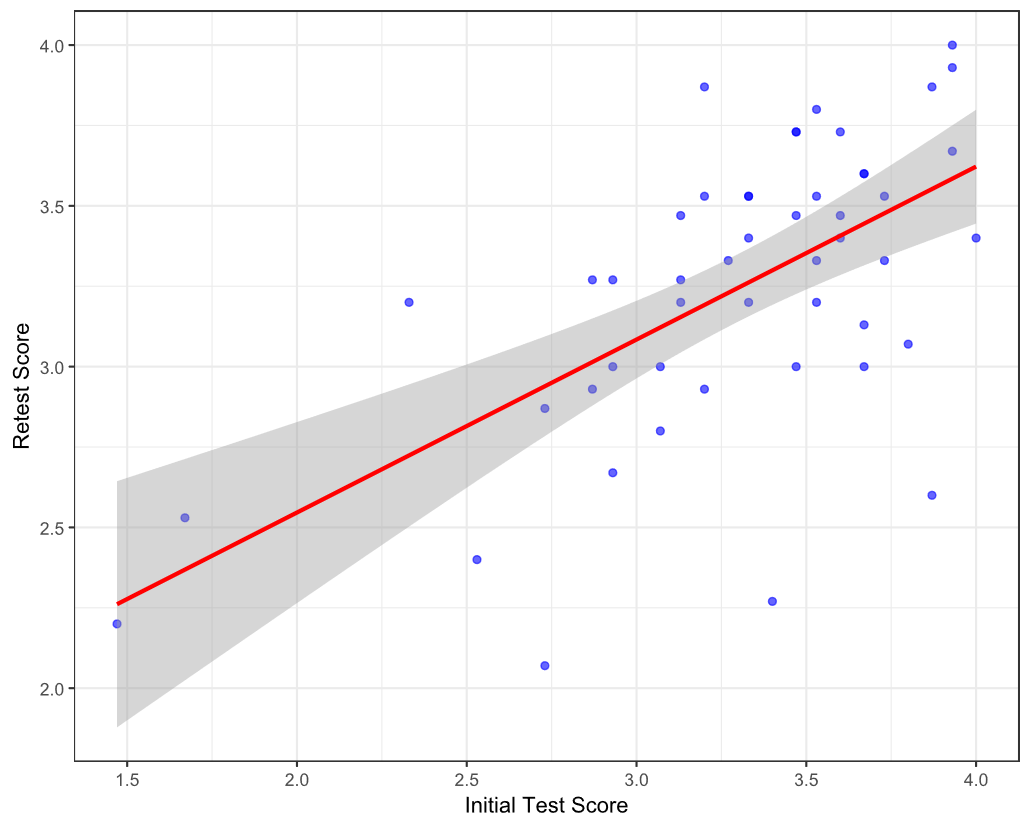


Grey shaded area represents the 95% confidence interval, which is approximated using the normal distribution of the standard error.

## Figure S1: Scatter plot showing test-retest scores of children completing the proposed HLS-Child-Q15-EN questionnaire with a 2 week interval


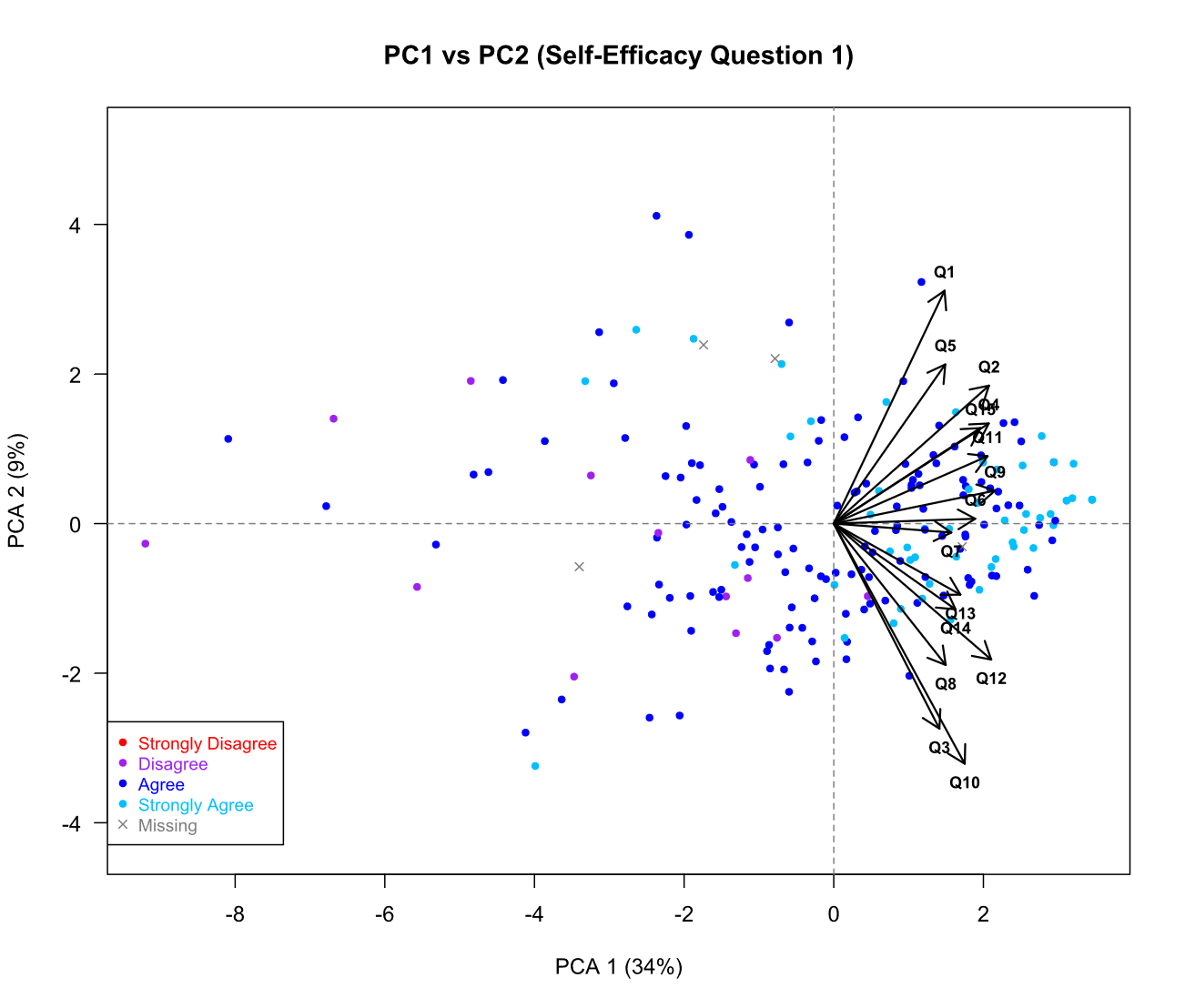


## Points represent values of observed data points in the principal component space. Arrows represent the loadings of each individual questionnaire item on PCA 1 and 2. Colour represents response to the first self-efficacy question, with greater agreement representing greater self-efficacy.

## Figure S2: PCA biplot of the first and second principal components. The first factor (PCA 1) represents a weighted average of the items, hence points with a large negative value in PC1 correspond to low scores in the HLS-Child-Q15. Opposing directions of rest-related (Q3, Q10) and illness-related (Q1, Q5) questions suggests that PCA 2 separates these two ideas – while PCA 1 may be related to “overall agreement” or overall health literacy, responses with larger positive values of PCA 2 suggest a strong understanding of health behaviours around illness and less understanding of health behaviours around rest, and vice versa.

# Supplementary tables

| **Number** | **Item** |
| --- | --- |
| Stem | How easy or difficult is it for you... |
| 1 | To find out how to get better from a cold quickly? |
| 2 | To find out what you can do to keep a healthy weight? |
| 3 | To find out how to relax best? |
| 4 | To find out which food is healthy for you? |
| 5 | To understand when and how to take your medications when you are sick? |
| 6 | To understand what a doctor is saying to you? |
| 7 | To understand why you need to see a doctor sometimes even though you are not sick? |
| 8 | To understand why you should have vaccinations? |
| 9 | To understand what your parents explain to you about your health? |
| 10 | To understand why you need to rest sometimes? |
| 11 | To decide which things are more healthy or less healthy? |
| 12 | To do what your parents tell you when you are sick so you can get better? |
| 13 | To take your medicine as you are told? |
| 14 | To remember the rules when you are crossing the road? |
| 15 | To eat healthy food? |

## Table S1: Items of the HLS-Child-Q15-EN after translation, qualitative pre-test, and consensus process by the research team

| **Questionnaire** | **NFI** | **RFI** | **IFI** | **TLI** | **CFI** | **RMSEA** |
| --- | --- | --- | --- | --- | --- | --- |
| HLS-Child-Q15 (German) | 0·869 | 0·819 | 0·912 | 0·867 | 0·910 | 0·044 |
| HLS-Child-Q15-EN | 0·787 | 0·743 | 0·870 | 0·840 | 0·867 | 0·077 |

NFI, Normed-Fit Index; RFI, Relative Fit Index; IFI, Incremental Fit Index; TLI, Tucker–Lewis Index CFI, Comparative Fit Index; RMSEA, Root Mean Square Error of Approximation.

## Table S2: Comparison of model fit indices for a three-factor model aligned to theoretical domains for the HLS-Child-Q15 (German) and HLS-Child-Q15-EN
