# Supplementary material for: Health literacy scale for English-speaking children: translation and validation of the HLS-Child-Q15-EN
Source: BMJ Open. 2025 Dec 17;15(12):e110215. doi: 10.1136/bmjopen-2025-110215 (PMC12716569; doi:10.1136/bmjopen-2025-110215)
Supplement: Supplementary file 2 [file bmjopen-2025-110215.reviewer_comments.pdf]

PEER REVIEW HISTORY

BMJ Open publishes all reviews undertaken for accepted manuscripts. Reviewers are asked to complete a checklist review form and are provided with free text boxes to elaborate on their assessment. These free text comments are reproduced below.

ARTICLE DETAILS

Title (Provisional)

A health literacy scale for English-speaking children: translation and validation of the HLS-Child-Q15-EN

Authors

Drake-Brockman, Thomas Frazer Eagle; Locke, Vance; Hauser, Neil; Sommerfield, David; Evans, Daisy; Sommefield, Aine; Khan, Nazim; von Ungern-Sternberg, Britta Sylvia

---

VERSION 1 - REVIEW

**Reviewer**	**1**
**Name**	**Izawa, Kazuhiro**
**Affiliation**	**Graduate School of Health Sciences, Kobe University**
**Date**	**16-Sep-2025**
**COI**	**None**

The purpose of the present study was to translate and validate the HLS-Child-Q15, a relatively short questionnaire for assessing health literacy in children originally validated in German, in English, to make it accessible to a large population of English-speaking children. After that author concluded that conducted a qualitative pre-test to assess comprehension and a validation to assess psychometric properties and test-retest reliability. This manuscript is written well; however, I recommend adding more study limitations in this manuscript.

**Reviewer**	**2**
**Name**	**Carrouel, Florence**
**Affiliation**	**Universite Claude Bernard Lyon 1**
**Date**	**21-Sep-2025**
**COI**	**None**

Dear authors,

This manuscript addresses an important gap: the lack of a validated health literacy questionnaire for English-speaking children. The study is well-designed, methodologically rigorous, and clearly reported. The translation process follows best practices, the validation sample is appropriate, and the results are consistent with prior studies.

Overall, the manuscript makes a meaningful contribution and should be of interest to both clinical and research audiences. However, some comments should be considered.

Major Comments

1. The ICC of 0.61 indicates only moderate reliability. While you acknowledge contextual differences between the hospital (initial survey) and home (retest), the discussion does not explore alternative explanations (e.g., cognitive development in children, response consistency, or situational variability).

Please expand on why reliability may be lower than expected and propose methodological improvements for future studies (e.g., standardizing administration setting, shorter retest intervals).

2. As in the original versions, no robust factor model was identified. The extracted factors and PCA largely reflected compliance/agreeability rather than theoretical domains.

Please discuss whether the HLS-Child-Q15 is inherently unidimensional, or whether multidimensionality cannot be captured with only 15 items. This is key for guiding users in interpreting scores.

3. Participants were recruited exclusively from a single institution (Perth Children's Hospital). While this is acknowledged, it limits the representativeness of English-speaking children globally (e.g., cultural/educational contexts in UK, USA, Canada).

Please state that further validation in other English-speaking populations is needed.

4. The study demonstrates internal consistency and known-group differences, but there is no external comparator (e.g., another established health literacy or reading comprehension tool).

Please add this as a limitation and clarify that future studies should evaluate criterion validity.

Minor Comments

5. The pre-test included only 10 children, which may be insufficient to fully capture linguistic diversity. Please justify why this number was chosen and highlight it as a limitation.

6. Some items were substantially reworded (e.g., "healthy food" instead of "healthy diet"). While these changes improve comprehension, they may reduce cross-cultural comparability. Please discuss potential impacts on comparability with German, Dutch, and French versions.

7. Children expressed a desire for a neutral option, but this was deliberately excluded to preserve comparability. This is a key methodological choice; please expand on why forcing a directional response is preferable.

8. The fit indices for the English version are lower than for the German version (CFI=0.867 vs 0.910, RMSEA=0.077 vs 0.044). The text briefly notes this but does not interpret it. Please elaborate on potential reasons and implications.

9. The discussion would benefit from a stronger emphasis on clinical/research applications. For example: in what contexts should clinicians use the tool (screening, baseline assessments, longitudinal follow-up)?

10. In the abstract and results, specify that the ICC indicates moderate (not high) reliability.

11. Figures (especially PCA biplot, Fig S2) could use more detailed captions to guide interpretation.

VERSION 1 - AUTHOR RESPONSE

Reviewer 1

The purpose of the present study was to translate and validate the HLS-Child-Q15, a relatively short questionnaire for assessing health literacy in children originally validated in German, in English, to make it accessible to a large population of English-speaking children. After that author concluded that conducted a qualitative pre-test to assess comprehension and a validation to assess psychometric properties and test-retest reliability. This manuscript is written well; however, I recommend adding more study limitations in this manuscript.

Thank you for your positive feedback. We have expanded the Limitations section, including in response to specific feedback from Reviewer 2.

Reviewer 2

This manuscript addresses an important gap: the lack of a validated health literacy questionnaire for English-speaking children. The study is well-designed, methodologically rigorous, and clearly reported. The translation process follows best practices, the validation sample is appropriate, and the results are consistent with prior studies. Overall, the manuscript makes a meaningful contribution and should be of interest to both clinical and research audiences. However, some comments should be considered.

Thank you for your positive feedback and detailed review. We have addressed each of the major and minor points raised below.

The ICC of 0.61 indicates only moderate reliability. While you acknowledge contextual differences between the hospital (initial survey) and home (retest), the discussion does not explore alternative explanations (e.g., cognitive development in children, response consistency, or situational variability).
Please expand on why reliability may be lower than expected and propose methodological improvements for future studies (e.g., standardizing administration setting, shorter retest intervals).

We agree, that only moderate reliability has been demonstrated, and this is stated in our Results and Discussion sections. We have expanded our comments on this in the Discussion section, including suggestions for further exploration.

As in the original versions, no robust factor model was identified. The extracted factors and PCA largely reflected compliance/agreeability rather than theoretical domains. Please discuss whether the HLS-Child-Q15 is inherently unidimensional, or whether multidimensionality cannot be captured with only 15 items. This is key for guiding users in interpreting scores.

Compliance is not necessarily disparate from literacy. For example, a child who understands the reason for taking medicine (e.g. to relieve pain) is more likely to do so even if they dislike the medicine.

Whilst the original scale was designed in a manner that might be expected to have multiple dimensions, these were not found in the German scale and are also not present in our translation.

We suggest that the HLS-Child-Q15-EN (and other versions) be used as a single output scale and would not recommend that groups of questions be extracted. Further work to explore the dimensionality of the scale may be warranted. We have noted these points in the Discussion section.

Participants were recruited exclusively from a single institution (Perth Children's Hospital). While this is acknowledged, it limits the representativeness of English-speaking children globally (e.g., cultural/educational contexts in UK, USA, Canada). Please state that further validation in other English-speaking populations is needed.

We have added this in our expanded Limitations section.

The study demonstrates internal consistency and known-group differences, but there is no external comparator (e.g., another established health literacy or reading comprehension tool). Please add this as a limitation and clarify that future studies should evaluate criterion validity.

We have added this in our expanded Limitations section.

The pre-test included only 10 children, which may be insufficient to fully capture linguistic diversity. Please justify why this number was chosen and highlight it as a limitation.

The sample of 10 children was chosen for pragmatic and resourcing reasons. We have clarified this in the Methods section. The interviewer was of the opinion that saturation was reached, and this is noted in the Results section. We have noted that seeking a greater variety (and therefore number) of participants may reveal further insights in the Limitations section.

Some items were substantially reworded (e.g., “healthy food” instead of “healthy diet”). While these changes improve comprehension, they may reduce cross-cultural comparability. Please discuss potential impacts on comparability with German, Dutch, and French versions.

We believe that the changes made will increase (rather than reduce) the comparability of responses, mitigating the impacts of cultural and language differences to a good degree.

In the specific case you reference, we note that the original German (dich gesund zu ernähren?) and translated Dutch (om gezond te eten?) refer to healthy (gesund/gezond) nourishment/eating (ernähren/eten). Both German and Dutch, unlike English, use a different word to refer to diet in the sense of “being on a diet” (Diät/dieet).

We have included additional discussion of this challenge, using Item 15 as an example, in the Discussion section.

Children expressed a desire for a neutral option, but this was deliberately excluded to preserve comparability. This is a key methodological choice; please expand on why forcing a directional response is preferable.

We feel that consistency with the original response options of the HLS-Q15-Child is sufficient justification for this choice. However, we also note that a neutral option can

be used by participants in a variety of different ways – e.g. as a true midpoint, or as an escape option for unfamiliar content – and this variability could be undesirable.

We have noted this additional factor in the Results section.

The fit indices for the English version are lower than for the German version (CFI=0.867 vs 0.910, RMSEA=0.077 vs 0.044). The text briefly notes this but does not interpret it. Please elaborate on potential reasons and implications.

The differences in fit indices are small and, therefore, we are confident that our results are sufficiently similar to that in the original paper. We have noted this in the Discussion section.

Further, while the fit indices have been performed for consistency of reporting, we feel that the manner in which these indices summarise a whole dataset down to a single number in a manner that is not overly useful.

The discussion would benefit from a stronger emphasis on clinical/research applications. For example: in what contexts should clinicians use the tool (screening, baseline assessments, longitudinal follow-up)?

Because of the short length of the HLS-Child-Q15-EN it is ideally suited to screening, or for assessment and follow-up of large cohorts. Because the tool is simple and short, it can be administered by the child themselves or with limited assistance by a parent. More detailed assessment of various contributing aspects of a child's literacy may be useful if a baseline is being measured prior to significant individual intervention, however this may be a more resources-intensive process.

We have noted these areas of utility in the Discussion section.

In the abstract and results, specify that the ICC indicates moderate (not high) reliability.

We do not claim high reliability anywhere in the abstract or results. We have added in the Abstract that the reliability was moderate, alongside the ICC of 0.61 which was already included. That test-retest reliability was moderate is already stated in the Results.

Figures (especially PCA biplot, Fig S2) could use more detailed captions to guide interpretation.

We have expanded the captions for Figure 2 and Figure S2, which now provide more guidance as to the interpretation of these figures.
